# A Honeycomb‐Structured Triboelectric Nanogenerator Based on Polyester Cloth for Smart Running Application

**DOI:** 10.1002/open.202400252

**Published:** 2025-01-27

**Authors:** Jianping Li, Zuhao Sun, Nan Wen, Yan Li

**Affiliations:** ^1^ Department of Physical Education and Research Lanzhou University of Technology Gansu China; ^2^ School of Physical Education Ningxia University Yinchuan China; ^3^ College of Physical Education YiLi Normal University Yining China; ^4^ Department of Pharmacy and Health Management Hebei Chemical & Pharmaceutical College Shijiazhuang China

**Keywords:** Triboelectric nanogenerators (TENGs), Self-powered sensor, Bio-mechanical energy harvesting, Intelligence sports, Running monitoring

## Abstract

Self‐powered devices for human motion monitoring and energy harvesting have garnered widespread attention in recent research. In this work, we designed a honeycomb‐structured triboelectric nanogenerator (H‐TENG) using polyester cloth and Teflon tape, with aluminum foil as the conductive electrode. This design leverages the large surface area and flexibility of textiles, resulting in significant performance improvements. The H‐TENG achieves an output voltage of 350 V, an output current of 42 μA, and a transfer charge (Q_SC_) of 77 nC, with a maximum output power of 465 μW. Additionally, the H‐TENG demonstrates the ability to monitor running activities and various gait patterns, providing real‐time bio‐mechanical data for smart running applications. The introduction of a stacked structure further enhances the output performance by increasing contact area and scalability, making the H‐TENG a robust and high‐performance energy harvester suitable for advanced wearable and flexible electronics.

## Graphical abstract

A honeycomb‐structured triboelectric nanogenerator (H‐TENG) is developed using textile‐based materials, achieving high output performance (Voc: 350 V, Isc: 42 μA, Qsc: 77 nC, power: 465 μW). The H‐TENG effectively monitors running activities and gait patterns, offering real‐time bio‐mechanical data. Its scalable stacked design enhances energy harvesting for advanced wearable and flexible electronics.

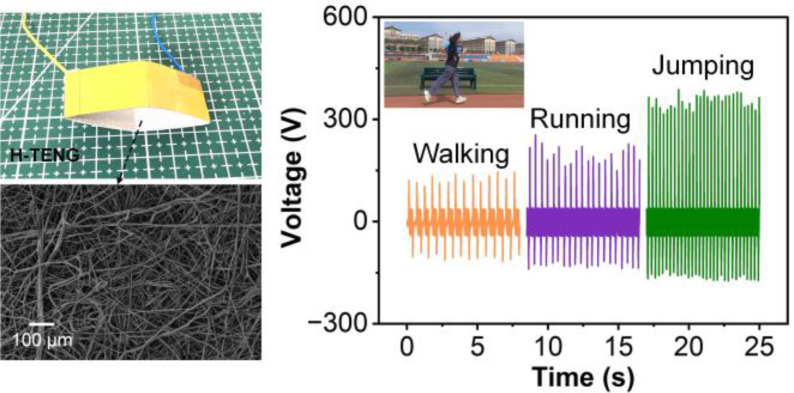

## 1 Introduction

With the continuous advancement of low‐power technology, nanoscale energy has emerged as a feasible means of providing power for small Internet of Things (IoTs) electronic devices.^[1]^ In this context, adopting emerging self‐powered technologies to meet the power demands of the IoTs has become increasingly viable.[^2^, ^3^] Self‐powered technology harnesses energy from IoTs electronic devices themselves or from the surrounding environment through various energy harvesting techniques, converting it into electrical energy.^[4]^ For example, in regions with abundant sunlight, solar cells based on the photovoltaic effect are well‐suited for collecting solar energy to power IoTs devices.^[5]^ Similarly, wind energy, which is prevalent in open mountainous areas, plateaus, and other regions, is a viable option for power generation.^[6]^ Additionally, the vast energy potential of water waves in the oceans, which cover 71 % of the Earth's surface, can be harnessed and converted into electricity to power IoTs devices operating at sea.[^7^, ^8^] However, these energy collection methods are limited by temporal and regional constraints, making them unsuitable for powering wearable IoTs devices. Currently, there is a significant gap in self‐powered technologies capable of supplying energy to wearable IoTs devices. In this context, flexible sensors have garnered considerable attention in the field of wearable devices due to their exceptional mechanical flexibility and strong adhesion properties.[^9^, ^10^] Flexible sensors can sense various motion through different mechanisms, including the piezoelectric effect,^[11]^ piezoresistive effect,^[12]^ capacitive effect,^[13]^ and thermoelectric effect.^[14]^ The piezoelectric effect relies on the principle of charge separation in materials under mechanical stress, but its output voltage and power density are relatively low, making it challenging to meet the demands of high‐energy‐consuming equipment.^[15]^ The piezoresistive effect involves signal conversion through changes in the resistance of materials under mechanical stress.^[16]^ Despite its high sensitivity, environmental temperature and humidity variations can impact its performance. The capacitive effect utilizes the change in capacitance value of a capacitor under pressure to detect external signals, but its signal conversion efficiency and stability require further improvement.^[17]^ The thermoelectric effect is based on the generation of electromotive force in materials subjected to a temperature gradient, yet the limited range of temperature variations in the human body results in low energy collection efficiency.^[18]^ Currently, the application of flexible sensors in wearable devices faces numerous challenges, including sensor stability, response rate, and more. Furthermore, existing self‐powered technologies encounter issues such as insufficient power density and unstable energy harvesting in practical applications. Therefore, future research should focus on optimizing the material and structural design of flexible sensors and integrating various energy harvesting mechanisms to enhance their performance and efficiency.

Textiles exhibit excellent mechanical flexibility and breathability, enabling seamless integration into triboelectric nanogenerators (TENGs) while ensuring comfortable, non‐irritating wear.[^19^, ^20^] Textiles have emerged as advantageous triboelectric materials for TENGs, offering unique benefits that make them highly suitable for flexible electronics and wearable devices.^[21]^ Compared to other triboelectric materials, textiles possess excellent mechanical flexibility, enabling seamless integration into flexible electronic and wearable devices while ensuring comfortable wear due to their soft texture and breathability.^[22]^ The structural diversity and designability of textiles allow customization of their electrical and mechanical properties, optimizing the triboelectric performance and energy harvesting efficiency of textile‐based TENGs through appropriate fiber selection and weaving techniques.[^23^, ^24^] Also, textiles’ large surface area and porous structure not only increase the triboelectric contact area and enhance triboelectric charge generation but also improve adsorption capacity, thereby boosting their ability to capture environmental energy. Textile‐based TENGs, easily integrated into everyday clothing without disrupting daily activities, are promising candidates for continuous energy support in portable electronic devices.^[25]^ Using natural fibers such as cotton and linen in textile TENGs offers environmental friendliness and sustainability due to their biodegradability and minimal environmental impact, aligning with green energy development goals.[^26^, ^27^] Compared to traditional rigid materials such as polymer films and metal foils, textiles provide superior mechanical flexibility, wearability, and structural diversity, while their porous and large surface area characteristics significantly enhance the energy harvesting capabilities of TENGs. Currently, TENGs are developed in various structural configurations, including Z‐shaped,^[28]^ rhombic,^[29]^ X‐shaped,^[30]^ and arched structure,^[31]^ each designed to optimize energy harvesting efficiency and adaptability. The Z‐shaped and X‐shaped structures enhance contact and separation processes, thereby increasing charge generation through improved surface interactions, while the rhombic structure offers robust stress distribution and high charge density. The arched structure leverages natural curvature to amplify contact area and mechanical deformation, providing structural stability and making it particularly effective for wearable and flexible electronics. Furthermore, stacked structure TENGs offer several advantages, such as increased surface area for charge transfer and enhanced energy output. This configuration involves layering multiple triboelectric pairs vertically or horizontally, thereby maximizing the effective contact area and enabling more efficient energy harvesting. Types of stacked TENGs include vertical stacking, where triboelectric layers are piled on top of each other, and horizontal stacking, where layers are arranged side by side.[^32^, ^33^, ^34^, ^35^, ^36^, ^37^] These designs allow for scalable energy output and improved mechanical robustness, making stacked TENGs highly suitable for applications requiring high power density and durability. Hence, integrating textile triboelectric materials with stacked TENG structures can significantly enhance energy harvesting efficiency by combining the large surface area and flexibility of textiles with the increased contact area and scalability of stacked configurations, leading to more robust, high‐performance energy harvesters for advanced wearable and flexible electronic applications.

Hence, we designed a honeycomb‐structured triboelectric nanogenerator (H‐TENG) using polyester cloth, bringing three significant advantages. The triboelectric pair in the H‐TENG consists of polyester cloth and Teflon tape, with aluminum foil serving as the conductive electrode. The honeycomb structure enhances the surface area and mechanical flexibility, improving energy harvesting efficiency.The integration of polyester cloth provides excellent durability and wearability, suitable for various physical activities. And the unique structural design allows for efficient bio‐mechanical energy conversion. The introduction of a stacked structure significantly improves the output performance of the H‐TENG by increasing the contact area and scalability, leading to higher energy harvesting efficiency. The H‐TENG achieves an output voltage of 350 V, an output current of 42 μA, and a transfer charge (Q_sc_) of 77 nC, with a maximum output power of 465 μW. Additionally, the H‐TENG demonstrates its capability in monitoring running activities and various gait patterns, providing valuable real‐time data for smart running applications. This innovation highlights the H‐TENG's potential for enhancing athletic performance and bio‐mechanical analysis through efficient energy harvesting and comprehensive motion monitoring.

## Experimental Section

### Materials

The substrate used was a flexible kapton film, chosen for its excellent mechanical properties, flexibility, and durability. The PET film provides a stable base for the other materials and ensures the overall robustness of the H‐TENG. High‐purity aluminum foil was utilized as the primary electrode material. Aluminum is selected for its superior electrical conductivity, ease of handling, and compatibility with the triboelectric effect. A finely woven polyester cloth was employed as the primary triboelectric material. Polyester is known for its positive triboelectric characteristics, making it an ideal choice for generating electrical charges upon mechanical interaction. Teflon (polytetrafluoroethylene, PTFE) tape was used due to its excellent triboelectric properties, particularly its high negative triboelectric potential.

### The Fabrication Process of H‐TENG Device

**Figure 1:**
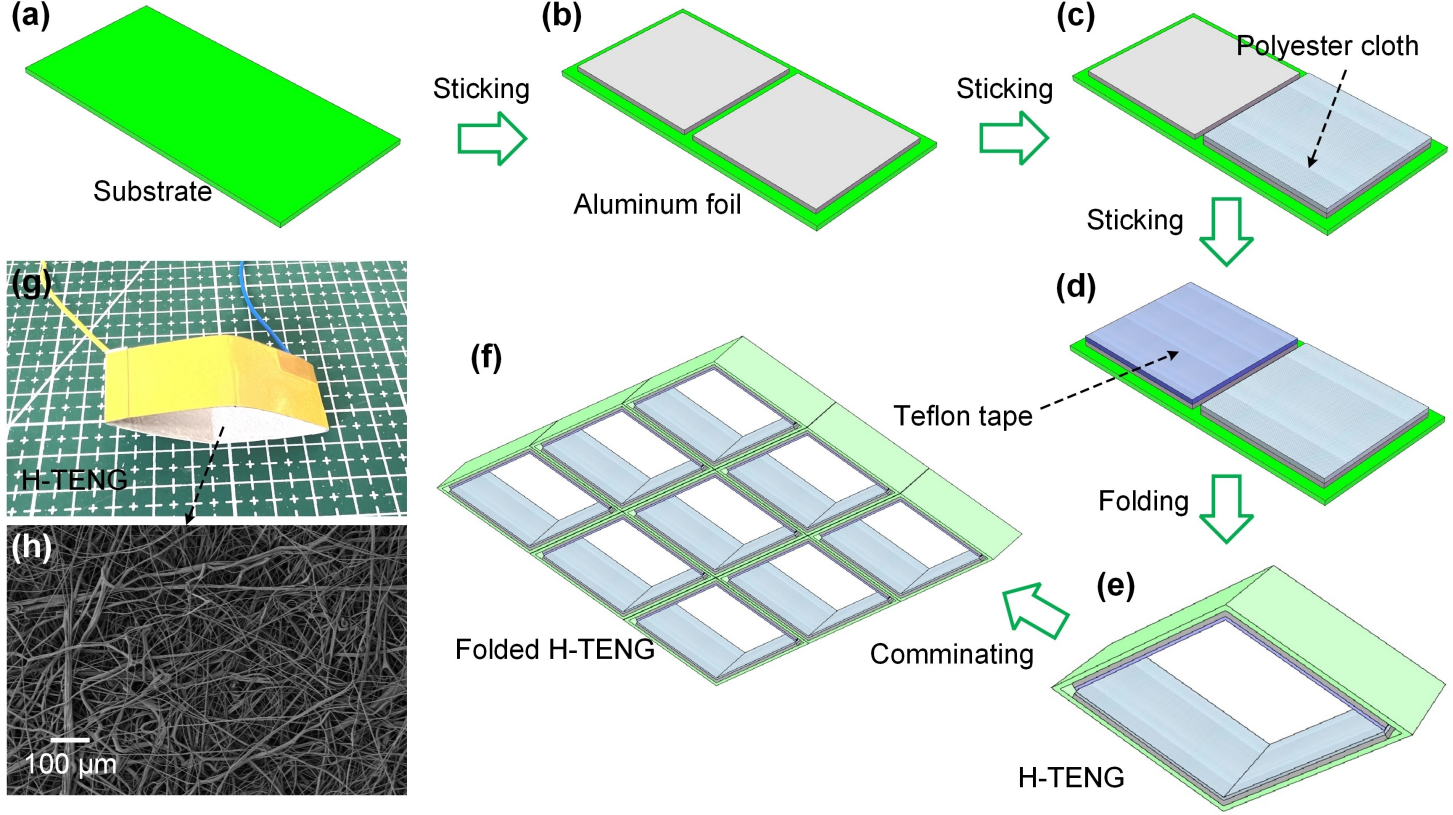
(a–e) Schematic illustration and fabrication process of the H‐TENG. (f) Schematic illustration of folded H‐TENG for increased output. (g) Photograph of the fabricated H‐TENG device. (h) SEM image of the polyester cloth's fibrous microstructure.

The preparation of H‐TENG involves a series of steps designed to fabricate a flexible, efficient triboelectric device. The process is illustrated in Figure 1(a–e), which is divided into several key stages. Initially, a flexible kapton substrate is prepared, which serves as the foundational layer for the H‐TENG device and provides structural support and facilitates the subsequent layers’ integration, as described in Figure 1(a). Next, a piece of aluminum foil is adhered to the substrate (Figure 1(b)). The aluminum foil acts as an electrode, crucial for the electrostatic induction effect. And this step ensures that the foil is firmly attached to maintain electrical conductivity and structural integrity. A layer of polyester cloth is then pasted over the aluminum foil surface (Figure 1(c)). The polyester cloth is chosen for its positive triboelectric properties, which are essential for generating electrical charges through mechanical interaction. The cloth must be uniformly adhered to avoid any inconsistencies in performance. Following the application of polyester cloth, Teflon tape is added (Figure 1(d)). Teflon tape, known for its high negative triboelectric potential, enhances the H‐TENG device's efficiency in generating electricity. Then fold the components together to form a compact diamond shaped structure (Figure 1(e)). To achieve higher energy harvesting efficiency, it is necessary to assemble multiple diamond shaped TENG arrays into a honeycomb structure TENG (Figure 1(f)).

### Characterization and Measurements

The completed H‐TENG unit is presented in Figure 1(g), highlighting its compact and flexible design. Additionally, the microstructure of the triboelectric material is illustrated in Figure 1(h), where a scanning electron microscope (SEM) image reveals the intricate fiber network of the polyester cloth, which is instrumental in the triboelectric effect. The meticulous preparation of H‐TENG involves substrate preparation, strategic layering of triboelectric materials, and precise folding to achieve a highly efficient and flexible energy harvesting device. The electrical performance of the H‐TENG was evaluated using a combination of a high‐precision electrometer (Keithley 6514) and a digital oscilloscope (RIGOL MSO5104). The Keithley 6514 was employed to measure the Q_sc_ and the output current generated by the H‐TENG. These measurements were conducted under controlled mechanical excitation to ensure consistent and repeatable results. In addition to these measurements, the output voltage waveform generated by the H‐TENG was captured using the digital oscilloscope. The oscilloscope provided detailed insights into the temporal characteristics of the voltage output, including the peak voltage, frequency response, and signal stability.

## 2 Results and Discussion

### 2.1 The Working Mechanism of H‐TENG Device

**Figure 2:**
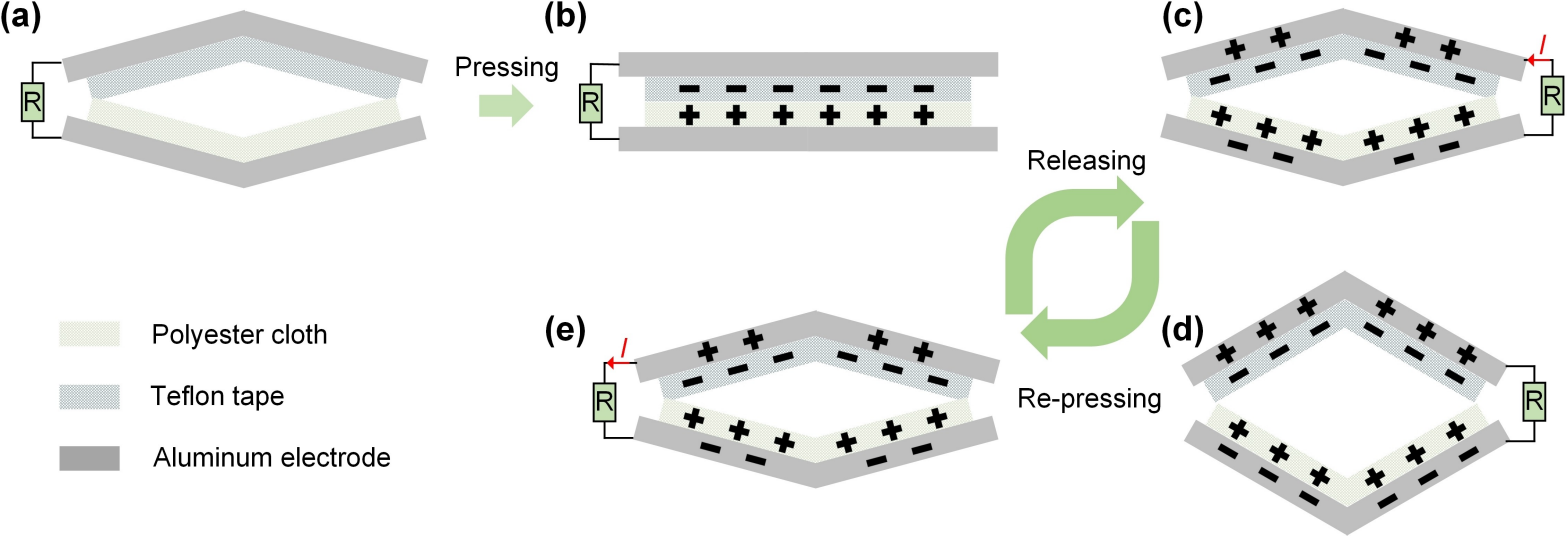
Working principle of H‐TENG. (a) Initial state with no applied force. (b) Pressing state where contact between polyester cloth and Teflon tape occurs, causing charge separation. (c) Releasing state where separated charges induce current flow through the external circuit. (d) The maximum separation state of triboelectric layers polyester cloth and Teflon tape. (e) Re‐pressing state initiating another cycle of charge transfer.

The working principle of the H‐TENG device is based on the contact‐separation mode. The process can be divided into several stages, as illustrated in Figure 2. In the initial state, there is no charges on Teflon tape and aluminum surfaces (Figure 2(a)). When a mechanical force is applied, the H‐TENG is pressed, causing the polyester cloth and Teflon tape to come into intimate contact (Figure 2(b)). During this contact, charge transfer occurs due to the triboelectric effect. Electrons are transferred from the polyester cloth to the Teflon tape, leading to a negative charge on the Teflon and a positive charge on the polyester cloth. This creates a potential difference between the aluminum electrodes connected to the polyester cloth and Teflon tape. Upon releasing the applied force, the H‐TENG structure returns to its original shape (Figure 2(c)). As the contact area between the polyester cloth and Teflon tape decreases, the separation of opposite charges induces an electric field, causing the electrons to flow through the external circuit from the negatively charged Teflon tape to the positively charged polyester cloth, generating an electric current. When the separation distance between Teflon tape and polyester cloth reaches its maximum, the output voltage of H‐TENG is highest, and the current in the external circuit is zero (Figure 2(d)). When the H‐TENG is pressed again, the process repeats. The contact between the cotton cloth and Teflon tape restores, leading to a reverse cycle of charge transfer and generation of electric current. The pressing and releasing cycles create an alternating current (AC) output. The H‐TENG can operate continuously through repeated pressing and releasing cycles. This repetitive mechanical motion causes continuous charge transfer and current generation, which can be harnessed for various energy harvesting applications.

### 2.2 The Electrical Output of H‐TENG Device

**Figure 3:**
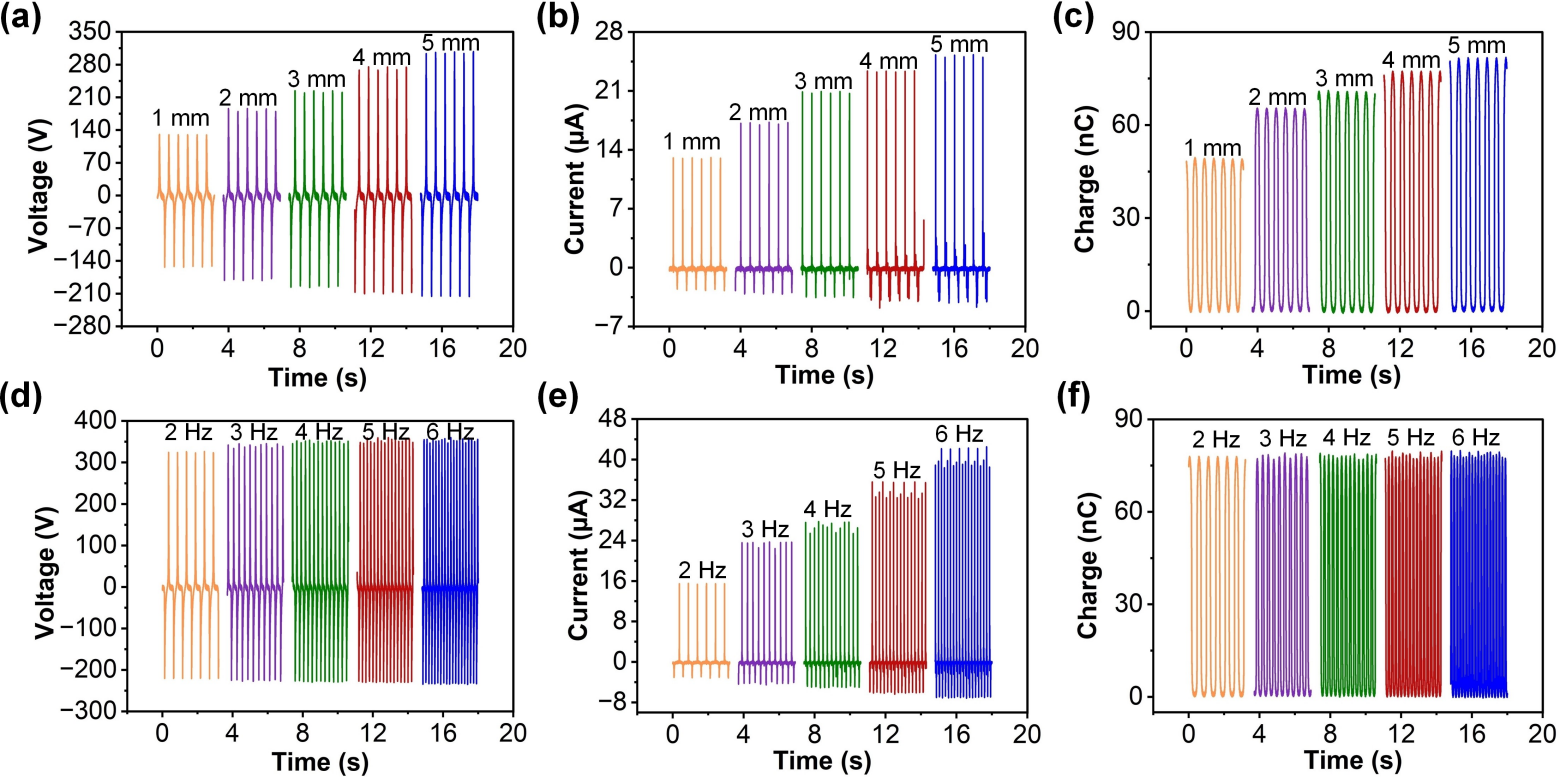
The performance of H‐TENG under varying separation distances and motion frequencies. The (a) output voltage, (b) output current, and (b) Q_sc_ of H‐TENG at different separation distances (1–5 mm). The (d) output voltage, (e) output current, and (f) Q_sc_ of H‐TENG at different motion frequencies (2–6 Hz).

The performance of the H‐TENG was systematically investigated by varying the maximum separation distance between the polyester cloth and Teflon tape surfaces, as well as the motion frequency. As illustrated as in Figure 3(a), the output voltage increased with the separation distance, reaching a peak value of approximately 308 V at a 5 mm separation, indicating that greater separation enhances the charge generation due to increased electrostatic induction. Similarly, the output current of H‐TENG exhibited an upward trend, with a maximum of about 25 μA at 5 mm. The increase in output current is attributable to the larger displacement allowing for more substantial charge transfer between the triboelectric layers. The Q_sc_ of H‐TENG also demonstrated a significant rise with increasing separation distance, peaking at around 81 nC at 5 mm, confirming that a larger separation enhances the effective contact area and thus the overall charge transfer (Figure 3(c)). Moreover, the output voltage of H‐TENG in Figure 3(d) remained 350 V across different frequencies, indicating that the H‐TENG can consistently generate high voltage under varying operational speeds. However, there is a slight increase in peak voltage as the frequency increases.The output current of H‐TENG in Figure 3(e) showed a noticeable rise with increasing frequency, reaching up to 42 μA at 6 Hz. This is likely due to the higher frequency leading to more frequent contact and separation cycles, thus enhancing the current output. The Q_sc_ peak value of H‐TENG maintained constant with frequency, similar to the output voltage, peaking at about 77 nC, as illustrated in Figure 3(f).

**Figure 4:**
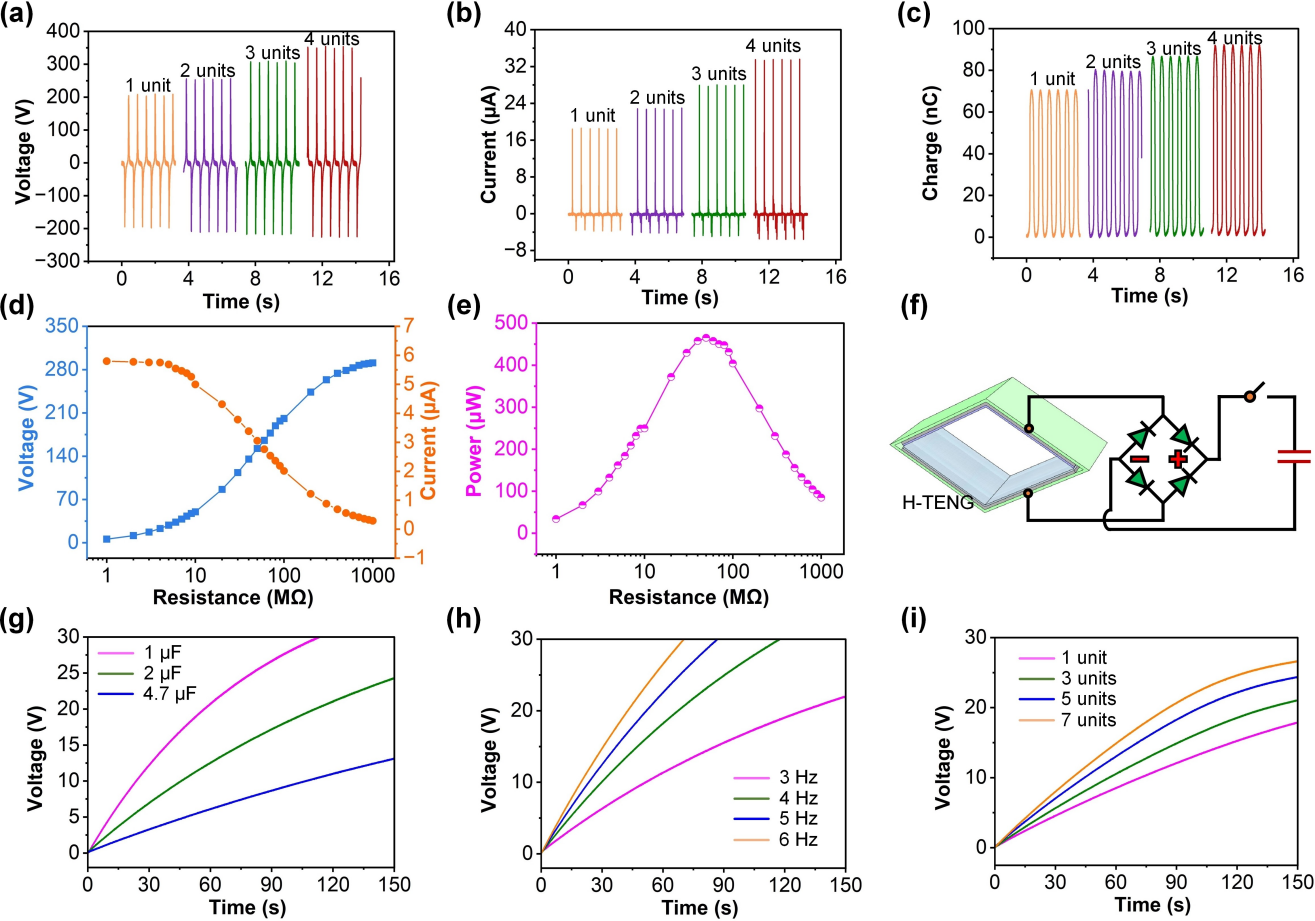
(a) output voltage, (b) output current, and (c) Q_sc_ of folded H‐TENG with different unit numbers (1–4 units). (d) Voltage and current of H‐TENG versus load resistance. (e) Power output of H‐TENG versus load resistance. (f) Schematic of the H‐TENG connected to a rectifier and capacitor. (g) Voltage across capacitors with different commercial capacitor (1 μF, 2 μF, 4.7 μF). (h) Voltage across a capacitor at different frequencies (3 Hz, 4 Hz, 5 Hz, 6 Hz). (i) Voltage across a capacitor with different unit numbers (1, 3, 5, 7 units).

The performance of the folded H‐TENG is significantly influenced by the number of working units. The output voltage of folded H‐TENG demonstrates a clear incremental trend with the addition of more units. Starting from approximately 209 V for a single unit, the voltage escalates to nearly 354 V with four units (Figure 4(a)). This enhancement is attributed to the cumulative effect of more triboelectric layers contributing to the overall charge generation. The output current of folded H‐TENG similarly increases with the number of units, reaching up to 33 μA for four units (Figure 4(b)). The rise in output current is due to the larger contact area and more significant charge transfer facilitated by the additional units. The Q_sc_ of folded H‐TENG also presents a proportional increase, peaking at around 92 nC for four units, indicating that the charge accumulation is directly influenced by the number of active triboelectric interfaces. The honeycomb structure significantly enhances the electrical output performance of the H‐TENG by integrating multiple independent cells into a compact and interconnected array. Each cell operates independently during the contact‐separation process, generating charges individually. The gaps between cells optimize the stress distribution, minimizing mechanical deformation in the contact regions. Additionally, the geometric characteristics of the honeycomb structure provide a larger effective contact area, leading to increased triboelectric charge generation and accumulation. The distributed design also reduces the impact of localized failures within a single unit, thereby improving the robustness and overall efficiency of the system. As the resistance increases from 1 MΩ–1000 MΩ, the output voltage of H‐TENG in Figure 4(d) exhibits a steady rise, whereas the output current decreases. This inverse relationship is typical in resistive loads, where higher resistance limits the current flow but allows a higher potential difference to build up. The power output of H‐TENG, calculated as P=V^2^/R, reaches a maximum at an optimal resistance of around 50 MΩ (Figure 4(e)). This peak power output of approximately 465 μW suggests that there is an ideal load resistance that maximizes the energy conversion efficiency of the H‐TENG. As demonstrated in Figure 4(f), the circuit configuration for capacitor charging involves rectifying the AC output of the H‐TENG using a bridge rectifier, subsequently storing the charge in a capacitor. When charging capacitors of different values (1 μF, 2 μF, and 4.7 μF), the voltage across the capacitors increases over time, as illustrated in Figure 4(g). Smaller capacitance values achieve higher voltages more rapidly due to lower energy storage requirements. The charging rate is also influenced by the operational frequency. Higher frequencies (up to 6 Hz) result in faster charging rates, as indicated by the steeper voltage‐time curves, as presented in Figure 4(h). This is because increased frequency leads to more frequent contact‐separation cycles, enhancing charge transfer. As shown in Figure 4(i), increasing the number of H‐TENG units accelerates the charging process. With more units, the voltage across the capacitor rises more quickly, highlighting the benefit of utilizing multiple H‐TENG units for efficient energy harvesting.

### 2.3 The Sensing Application of H‐TENG Device on Running Motion

**Figure 5:**
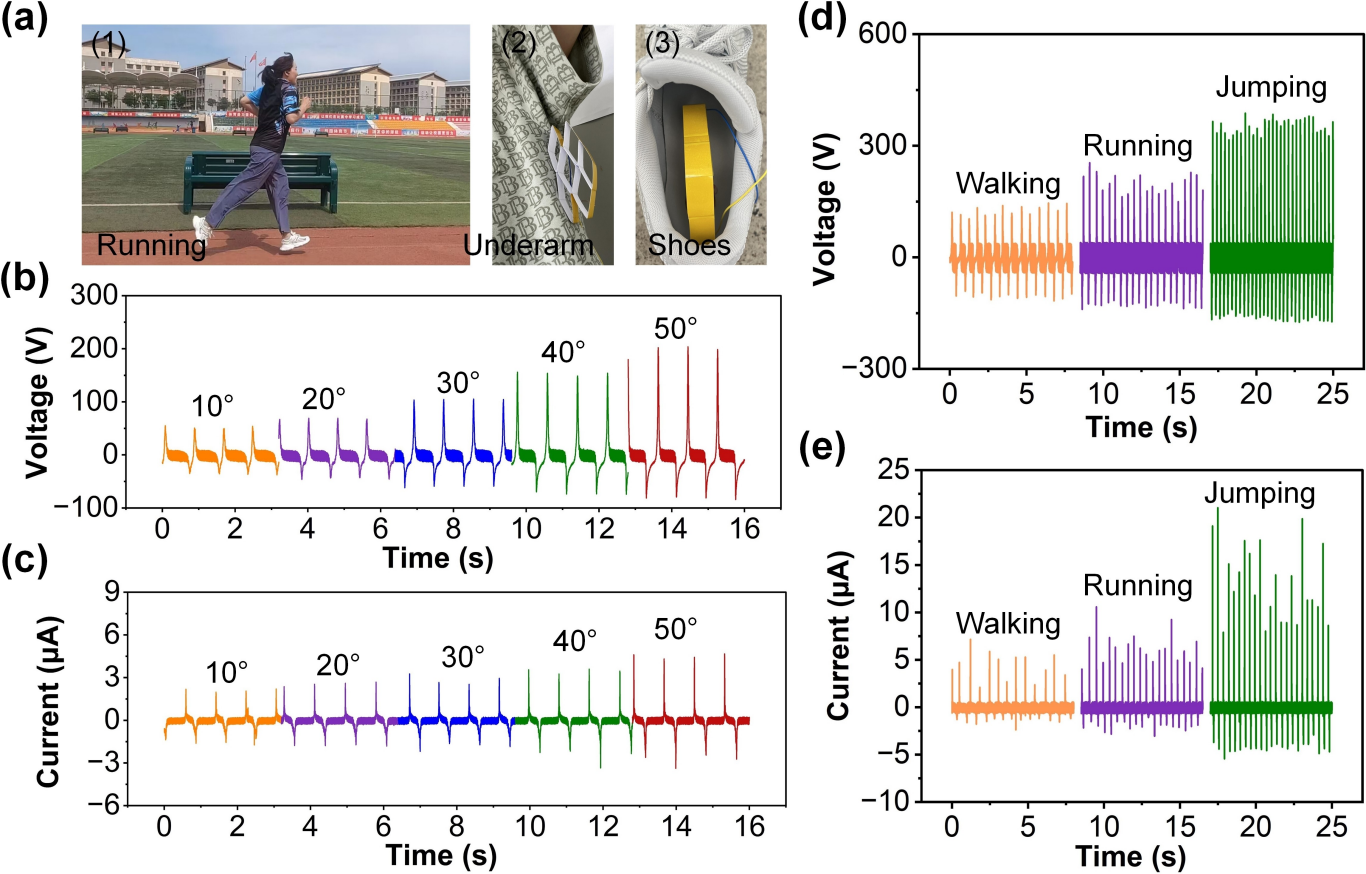
Application and performance of H‐TENG for real‐time monitoring in running sports. (a) Application scenarios: (1) Running; (2) Underarm placement for monitoring arm swing; (3) Shoe integration for gait analysis. (b) Voltage output and (c) current output of H‐TENG at different arm swing angles (10°, 20°, 30°, 40°, and 50°). (d) Voltage output and (e) current output during different activities: walking, running, and jumping.

The monitoring effect of a single‐unit H‐TENG compared to a multi‐cell honeycomb structure can be understood from their intrinsic design differences. A single‐unit configuration, while capable of generating electrical signals during motion, is limited in terms of sensing resolution and signal stability due to its smaller effective contact area and less uniform stress distribution. Conversely, the honeycomb structure, composed of multiple independent cells, significantly enhances these aspects by increasing the overall contact area and uniformly distributing the applied mechanical stress. This ensures that motion signals are captured more effectively and consistently across the H‐TENG device. Figure 5(a1–a3) illustrates the application of H‐TENG for real‐time monitoring of an athlete's arm swing amplitude and gait information, providing valuable data for smart sports. Figure 5(b and c) present the voltage and current outputs corresponding to different arm swing angles (10°, 20°, 30°, 40°, and 50°). The results indicate a clear correlation between the arm swing amplitude and the generated electrical signals, with higher angles producing higher voltages and currents. Figure 5(d and e) show the voltage and current outputs during different physical activities, including walking, running, and jumping. The voltage and current signals vary significantly with each activity, demonstrating the H‐TENG's capability to distinguish between different types of movement and providing detailed information about the athlete's gait patterns. The application of H‐TENGs in smart sports enables real‐time bio‐mechanical monitoring and energy harvesting, providing valuable data for optimizing athletic performance and injury prevention. This integration enhances training efficiency and supports the development of personalized, adaptive training programs in smart sports environments.

## 3 Conclusions

In conclusion, the H‐TENG designed with polyester cloth and PTFE tape, and utilizing aluminum foil as the conductive electrode, demonstrates significant advancements in energy harvesting and bio‐mechanical monitoring for smart running applications. The unique combination of a honeycomb structure and stacked configuration significantly enhances the surface area, contact efficiency, and overall energy output. The H‐TENG achieves an open‐circuit voltage of 350 V, a short‐circuit current of 42 μA, and a transfer charge of 77 nC, with a maximum output power of 465 μW. These impressive performance metrics highlight its potential for providing continuous energy support and detailed real‐time motion data, thereby optimizing athletic performance and aiding in injury prevention. Future research will focus on further optimizing the material composition and structural design to enhance the efficiency and applicability of H‐TENGs in various wearable and flexible electronic applications.

## Conflict of Interests

The authors declare no conflict of interest.

4

## Data Availability

Data available on request from the authors.
